# Phenological overlap of interacting species in a changing climate: an assessment of available approaches

**DOI:** 10.1002/ece3.668

**Published:** 2013-07-22

**Authors:** Nicole E Rafferty, Paul J CaraDonna, Laura A Burkle, Amy M Iler, Judith L Bronstein

**Affiliations:** 1 Department of Ecology and Evolutionary Biology, University of ArizonaTucson, Arizona, 85721; 2 Center for Insect Science, University of ArizonaTucson, Arizona, 85721; 3 The Rocky Mountain Biological LaboratoryCrested Butte, Colorado, 81224; 4 Department of Ecology, Montana State UniversityBozeman, Montana, 59717; 5 Department of Biology, University of MarylandCollege Park, Maryland, 20742

**Keywords:** Climate change, community, demography, experiment, life history, long-term data, models, observation, phenology, simulation

## Abstract

Concern regarding the biological effects of climate change has led to a recent surge in research to understand the consequences of phenological change for species interactions. This rapidly expanding research program is centered on three lines of inquiry: (1) how the phenological overlap of interacting species is changing, (2) why the phenological overlap of interacting species is changing, and (3) how the phenological overlap of interacting species will change under future climate scenarios. We synthesize the widely disparate approaches currently being used to investigate these questions: (1) interpretation of long-term phenological data, (2) field observations, (3) experimental manipulations, (4) simulations and nonmechanistic models, and (5) mechanistic models. We present a conceptual framework for selecting approaches that are best matched to the question of interest. We weigh the merits and limitations of each approach, survey the recent literature from diverse systems to quantify their use, and characterize the types of interactions being studied by each of them. We highlight the value of combining approaches and the importance of long-term data for establishing a baseline of phenological synchrony. Future work that scales up from pairwise species interactions to communities and ecosystems, emphasizing the use of predictive approaches, will be particularly valuable for reaching a broader understanding of the complex effects of climate change on the phenological overlap of interacting species. It will also be important to study a broader range of interactions: to date, most of the research on climate-induced phenological shifts has focused on terrestrial pairwise resource–consumer interactions, especially those between plants and insects.

## Introduction

Shifts in the timing of life-history events are among the strongest biological signals of anthropogenic climate change (Root et al. 2003; Cleland et al. 2007). Although the magnitude and direction of phenological responses vary among species, the general trend is for springtime life-history events to shift earlier, consistent with warming temperatures (Menzel et al. 2006; Parmesan 2007; Cook et al. 2012). For example, flowering onset, butterfly emergence, and migratory bird arrival to breeding sites have all advanced at many locations (Visser et al. 1998; Fitter and Fitter 2002; Stefanescu et al. 2003).

Phenological shifts can have direct effects on species by exposing individuals to unfavorable abiotic conditions, such as early season frost in the temperate zone (Inouye 2008; Thomson 2010). Phenological shifts can also affect the nature and strength of interspecific interactions (Burkle et al. 2013). They can alter the relative timing of life-history events, changing the extent of temporal overlap with mutualists and antagonists (Parmesan and Yohe 2003; Hegland et al. 2009; Potts et al. 2010), ultimately leading to changes in demographic processes (Miller-Rushing et al. 2010; Boggs and Inouye 2012; but see Reed et al. 2013).

Whereas earlier work on the temporal overlap of interacting species focused on adaptive processes shaping the evolution of phenological patterns (Waser 1979; Augspurger 1981; Brody 1997), recent interest has shifted to consider how anthropogenic climate change–induced phenological shifts will affect the ecology of interactions. Although this is a relatively new question, the effects of phenological shifts on interacting species are of great concern, both for the persistence of individual species and for the provision of ecosystem services (Hegland et al. 2009). In many cases, it is only by understanding how shifts in phenology affect species interactions that the consequences of these shifts can be forecasted. As Visser and Both (2005) argued, it is difficult to know whether a change in phenology is likely to have a negative, neutral, or positive effect without a ‘yardstick’ that is meaningful for the species of interest. In many instances, meaningful yardsticks involve the timing of interactions and phenological matching with other species. For example, to understand how earlier egg laying will affect bird populations, it is important to know how the shift affects synchrony with peak biomass of prey (Visser and Both 2005).

Given that it is often necessary to study interactions to understand the impacts of climate change–induced shifts in phenology, it is worthwhile to consider how information on the phenological overlap of interacting species is obtained and the questions that different approaches can answer. As research at the intersection of climate change, phenology, and species interactions has expanded, so too have the ways in which researchers investigate these phenomena. A multitude of approaches are in use, ranging from observational methods that take advantage of natural variation in phenology, to experimental studies focused on causation, to abstract models that may or may not generate testable predictions. Researchers focused on different types of interactions tend to use different approaches. To date there has been no overview or evaluation of approaches across subdisciplines.

Here, we define the key questions driving the study of the phenological overlap of interacting species in the context of climate change, and survey the literature to evaluate current approaches to address them. Much of the research on this topic has been shaped by the availability of preexisting data. Now that the groundwork has been laid, researchers have the opportunity to be more deliberate about how they approach these questions. To help foster such a shift, we present a conceptual framework centered around key research questions and approaches. We then explore the merits and limitations of each approach, using examples from the literature to illustrate their utility. We also survey the published literature to quantify the frequency with which each approach is being used and to characterize the types of interactions that have been studied. Finally, we evaluate some of the challenges and profitable directions for future research on climate-induced phenological shifts and species interactions, and highlight the value of combining methods.

## Conceptual Framework

Spanning much of the research in this field is one central question: *What are the consequences of phenological shifts for species and their interactions?* Few studies have been able to directly address this question (but see, e.g., van Asch et al. 2007; Fabina et al. 2010; Liu et al. 2011), largely because of a scarcity of data on the demographic and fitness consequences of changes in phenology (Miller-Rushing et al. 2010). Instead, recent studies attempt to answer one or more of three narrower questions. First, *how is* the phenological overlap of interacting species changing (if at all) as a result of climate change? Second, *why is* the phenological overlap of interacting species changing in a mechanistic sense? Finally, *how will* the phenological overlap of interacting species change in the future, given assumptions about the trajectory of climatic changes and the plasticity of interspecific interactions?

We can identify five approaches for studying climate-driven phenological shifts and species interactions: (1) interpretation of long-term phenological data, which relies on time series of adequate duration to allow comparisons between past and present phenologies; (2) field observations, which take advantage of phenological variation over shorter timescales; (3) experimental manipulations, which enable researchers to directly measure the consequences of altering the phenologies of study organisms; (4) simulations and nonmechanistic models, which generate predictions under future climate change scenarios; and (5) mechanistic models, which shed light on the cues and triggers that shape phenologies. Variables quantified with these approaches include extent of temporal overlap of interacting species, ideally measured in terms that are biologically meaningful for the species of interest (Visser and Both 2005), interaction frequency, fitness measures, demographic parameters, and population persistence.

**Figure 1:**
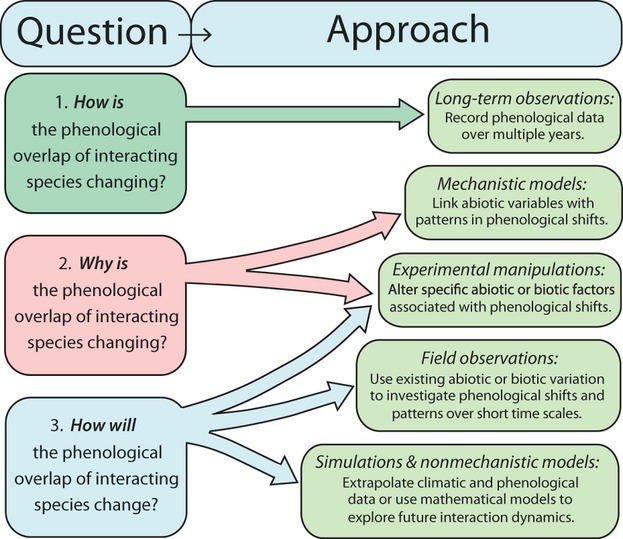
Conceptual framework connecting three key questions driving research on the effects of anthropogenic climate change on the phenological overlap of interacting species to five common approaches described in the text.

Figure 1 presents a conceptual framework that illustrates the connections among the three questions we define above and the approaches used to address them. As Figure 1 indicates, a limited number of approaches can answer each question. Therefore, if the goal is to answer all three questions, then multiple approaches are essential. If, for instance, a study seeks to determine both how and why the phenological overlap of interacting species is changing as a result of climate change, then long-term phenological data must be combined with either experimental manipulations or mechanistic models. Below, we examine the literature to describe which types of interactions are being studied with these approaches. We then discuss each approach in turn, before elaborating on fruitful combinations of approaches indicated in Figure 1.

## Literature Survey

As a basis for our discussion of research trends, we surveyed the literature on how climate change–driven shifts in phenology affect species interactions, with a focus on the approaches used in each study. A Web of Science search criterion of “interact* AND phenolog* AND climate change” spanning the years 1995–2012 yielded 422 papers. We started our search with the year 1995 in part because this is when research on this topic began to expand; our search returned no studies for the prior year and only four studies for the years 1990–1993. Although our search was not all inclusive, it provided an unbiased sample of the literature from which key trends could be detected. Each study was examined to determine whether it included information on phenological shifts attributed to climate change and the resulting effects of shifts on species interactions. We focused on the 82 studies that met these criteria.

We categorized these studies based on: (1) general category of interaction (e.g., mutualism), (2) specific type of interaction (e.g., plant–pollinator mutualism), and (3) habitat (e.g., terrestrial). As Table 1 shows, almost 90% of the interactions studied to date have been either mutualistic or antagonistic resource–consumer interactions. Most of these interactions are predator–prey, plant–herbivore, and plant–pollinator interactions; indeed, almost all studies on mutualism are on plant–pollinator interactions (Table S1). Only 11% of studied interactions involve competition (Table 1). Two thirds of studies have been conducted in terrestrial habitats and 40% involve vertebrates (Table S1). Finally, we categorized studies based on approach to explore how frequently each approach is used and whether some interactions tend to be studied with certain approaches (Table 1). We detail these findings as we consider the approaches in turn below. The remainder of this study uses this literature as a basis for weighing the strengths and weaknesses of each approach and pointing to future challenges.

**Table 1:** Results of a literature survey of studies that address the effects of climate change on the phenological overlap of interacting species

Approach	Interaction			

	All	–, −	+, −	+, +
Interpretation of long-term data	98 (59%)	10	76	12
Field observations	9 (5%)	0	8	1
Experimental manipulations	18 (11%)	4	9	5
Simulations and nonmechanistic models	11 (7%)	1	5	5
Mechanistic models	30 (18%)	4	24	2
Total	166	19 (11%)	122 (74%)	25 (15%)

The 82 studies we identified investigated 166 interactions. We categorized each interaction (denoted −, − for competitive; +, − for antagonistic resource–consumer; and +,+ for mutualistic interactions) according to the approach(es) used.

## Approaches

### Interpretation of long-term phenological data

#### Overview

Long-term phenological data on life-history events such as leaf budburst, flowering, insect emergence, egg laying, and plankton blooms were used in the study of nearly 60% of the interactions in our survey (Table 1). For this analysis, we define “long-term” as time series of at least 6 years. Although we recognize that what constitutes long term depends on the natural history of the organism, a 6-year minimum encompasses studies using historical data that span decades, as well as studies using contemporary data on organisms for which historical data may not exist. These data offer unique insight into the question: How is the phenological overlap of interacting species changing as a result of climate change (question 1, Fig. 1)? For example, data on the laying dates of birds and timing of peak biomass of their insect prey collected over multiple decades revealed that temporal synchrony between predators and prey has decreased (Visser et al. 1998). With concurrent climate data, long-term records can also provide insight into the mechanisms of phenological shifts to address the question: Why is the phenological overlap of interacting species changing (question 2, Fig. 1)? In combination with models, long-term phenological data can help answer the question: How will the phenological overlap of interacting species change in the future (question 3, Fig. 1)? However, which questions can be answered depends in part on the temporal scale and resolution of long-term data sets, which can vary tremendously. Ideally, records are collected systematically, with repeated sampling at regular intervals in the same location. Nevertheless, data collected in a piecemeal fashion can be valuable; for example, observations by citizen scientists and information gleaned from photographs and museum specimens have documented phenological shifts (Roy and Sparks 2000; Miller-Rushing et al. 2006).

#### Examples

Long-term data have been used to address phenological shifts in a wide variety of interactions, both terrestrial and aquatic, and both mutualistic and antagonistic (Table 1 and S1). Edwards and Richardson (2004) have presented an exemplary study that used long-term data to investigate climate change–induced phenological shifts in a marine system in the North Sea. Using a data set spanning more than 40 years, they show that the timing of seasonal peaks in abundance has been affected differently by climate change for three trophic levels of plankton, resulting in temporal mismatches that could cascade up the food chain to economically important fish species. The phenology of some plankton groups remained relatively constant, whereas others displayed large temporal shifts, resulting in loss of synchrony among three levels of production. These responses were then related to abiotic factors, including temperature, to gain some understanding of the mechanisms at work. Thus, long-term data in this case provided insight into not only how but also why the phenological overlap of these interacting species is changing as a result of recent warming.

#### Merits

With long-term records, it is possible to determine whether different species show similar responses to changing environmental cues, regardless of what those cues may be. Assuming that the data are reliable, this approach is likely the most accurate at capturing shifts in the timing of life-history events, especially if preclimate change data can be obtained that establish the prior phenological overlap of interacting species. If many years of data are available, then it is also possible to detect the trajectory of the phenological response to climate change, which may be nonlinear (Inouye 2008). Such information can, in turn, inform models that seek to predict future responses to climate change. Lastly, in identifying which species are experiencing phenological shifts and in quantifying the varying magnitudes and directions of those shifts, long-term data can reveal broad patterns and motivate further study of particular species or interactions with other approaches.

#### Limitations

Inherent in the use of long-term data is the assumption that records are of adequate duration to detect phenological shifts or to capture the true preclimate change baseline of phenological synchrony (Singer and Parmesan 2010). If a time series falls within a period of anomalous climate conditions, phenological shifts might be obscured or inflated. Indeed, there can be substantial variation in both the direction and magnitude of phenological shifts within temporal subsets of long-term records (Iler et al. in press). In most cases, data are available only for the initiation of phenological phases, and the accuracy of such observations can be influenced by sampling frequency and population size (Miller-Rushing et al. 2008). Limiting the use of this method rather than its utility is the simple fact that historical records are rare or nonexistent for many organisms, particularly those that are mobile and inconspicuous, and for many geographical locations; our survey found a strong bias toward terrestrial systems, and no long-term records for tropical ecosystems (Table S1). Along similar lines, because temporal events can be autocorrelated, analysis of long-term data may require time-series statistics with which many ecologists are unfamiliar.

### Field observations

#### Overview

Short-term observational data that take advantage of natural within-season variation in the timing of life-history events can reveal how individuals that differ in phenology are likely to be affected under different climate change scenarios. Along the same lines, extreme interannual weather events that alter phenology can serve as opportunistic events (sometimes termed natural experiments) that provide a way to test whether interacting species will respond similarly to climatic changes. These approaches by themselves can be used to address only the third question in Figure 1: How will the phenological overlap of interacting species change? They cannot fully unveil the mechanisms of phenological shifts (question 2), although they can hint at which cues are important over short timescales. Nor can they reveal how the phenological overlap of interacting species is changing (question 1), unless long-term or historical data are available for comparison. The weather events that permit natural experiments can be brief, spanning only a few weeks (e.g., a summer heat wave), or sustained, extending over an entire season (e.g., a summer drought). Interactions shaped by an extreme weather event are usually compared with those in years of typical (i.e., closer to the mean) conditions to test for differential responses. Even without extreme weather, however, short-term field observations can be informative. For instance, the timing of senescence among plants could be correlated with herbivore activity to elucidate how each partner would be affected by climatic changes that shift senescence earlier or later within the observed phenological range.

#### Examples

Surprisingly few studies have used short-term field observations (Table 1). Wall et al. (2003) demonstrated that the endangered plant *Clematis socialis* was visited by different insect pollinators depending on whether flowering occurred early in an unusually warm year or late in an especially cool year. Thus, the pollinators did not respond in the same way as the plant to springtime temperature fluctuations in this natural experiment, addressing the question: How will the timing of interactions change? Because the primary pollinator in the warm year made fewer visits to *C. socialis* and tended to be less effective, the reproductive output of the plant was likely lower, addressing the consequences of altered phenology. Using short-term observations of natural variation in flowering time within individuals of *Mertensia fusiformis* to address these same questions, Forrest and Thomson (2010) were able to show that later flowers could reduce the likelihood of reproductive failure if flowering were to begin before pollinators were active.

#### Merits

A strength of these approaches is that all species in the community are exposed to the same climatic conditions and cues (although species might experience those cues differently). Therefore, their responses should provide accurate information about whether the same abiotic factors will affect their phenologies similarly. By taking advantage of opportunistic events, we might also gain information on how interactions will be affected by increased variability in climatic conditions. Increased variance in temperature and precipitation might have stronger effects on ecological systems than a slowly shifting mean (Crimmins et al. 2011), making the collective insight provided by these opportunistic events crucial to understanding the effects of phenological change on community structure and function. Finally, short-term observations of phenological variation can be a practical alternative to experiments, particularly when data are collected along an environmental gradient, and can develop into longer term and/or historical records.

#### Limitations

A fundamental weakness of opportunistic events is that they are impossible to replicate and difficult to anticipate. However, researchers may have preexisting data collected under typical versus atypical weather conditions that could be compared. Also, because the value of short-term observation hinges on phenological variation within or among populations, this approach is of limited utility in species with highly synchronous phenologies.

### Experimental manipulations

#### Overview

Direct experimental manipulation of phenology is a powerful way to understand how interacting species will respond to climatic changes that shift phenology beyond the current range of variation (question 3, Fig. 1). To illuminate how changes in timing will affect the strength or nature of an interaction, environmental conditions can be manipulated to alter phenology in certain directions (Rafferty and Ives 2011). In addition to addressing how the phenological overlap of interacting species will change in the future, experiments can elucidate the underlying mechanisms of phenological responses to reveal why the phenological overlap of interacting species is changing (question 2, Fig. 1). To uncover the mechanisms underpinning phenological responses to climate change, one can directly alter abiotic cues and measure the responses (Keller and Körner 2003). Reciprocal transplants can also be carried out to determine, for example, the extent to which phenologies are genetically versus environmentally determined along an elevational gradient (Clausen et al. 1941; Forrest and Thomson 2011).

#### Examples

Three recent studies have used an exclusively experimental approach to manipulate plant phenology and measure the consequences for interactions with mutualistic and/or antagonistic insects. To alter flowering phenology in calcareous grasslands, Parsche et al. (2011) raised plants under different greenhouse conditions, whereas Warren et al. (2011) transplanted plants to north- or south-facing slopes in deciduous forest. Liu et al. (2011) directly manipulated both plant and insect herbivore phenology, using open-top chambers to warm plants and moth larvae in an alpine meadow. This experimental approach revealed that the direction of the warming effect differed between interacting partners: in the plants, peak flowering and aboveground senescence occurred earlier, whereas in the insects, larval emergence was delayed. This shift in relative timing affected interaction strength, with large negative consequences for the reproductive output of one of the plant species. Thus, the authors gained insight into how the phenological overlap of interacting species will change, as well as the potential consequences of such change.

#### Merits

An obvious strength of experimental approaches is that they allow direct manipulation of both the magnitude and direction of induced shifts in phenology. It follows that this approach can allow researchers to replicate phenological shifts and to control abiotic and biotic variables fairly precisely. Another advantage is that experiments can negate the temporal autocorrelation inherent in long-term and observational data; a consistent response across time and space is strong evidence that the phenological manipulation is the driver. Experimental manipulations are unique in enabling researchers to dictate the exact timing, duration, and magnitude of interactions between species. Fitness consequences of interacting at different points along the phenological trajectories of species can thereby be measured (Yang and Rudolf 2010). Finally, experiments can disentangle how potentially interacting climate factors, such as temperature, carbon dioxide, and precipitation might combine to affect phenology and, consequently, species interactions (Hoover et al. 2012).

#### Limitations

A basic limitation of the experimental approach is the impracticality of manipulating the phenologies of organisms that are difficult to observe, have slow life histories relative to our own, or occur at low population densities. The subjects of the experimental studies in our survey were almost always plants and/or insects (Table S1). Another potential weakness of directly manipulating phenologies lies in the risk that ancillary traits are affected in ways that may not reflect the effects of climate change. Plants forced to flower at different times, for example, may differ in nectar volume and sugar content (Rafferty and Ives 2011). Controlling for variation in all these additional traits is often unfeasible, yet they may affect interactions and should therefore be acknowledged. In addition, if the phenology of only one species is manipulated, as is almost always the case to date, then interpreting the effects on interactions can be problematic because other species will not have experienced the same cues. For instance, if a plant is forced to germinate early in a greenhouse and is then exposed to herbivorous insects in the field, those insects will not have experienced the cues that, under natural conditions, would have caused the advanced phenology of the plant. Thus, a caveat of this approach is that the results can represent a worst case scenario in which the potential for mismatch is likely maximized. Along similar lines, it is usually practical to directly alter only a single phenological event, even though climate change is likely to affect a variety of phenophases and hence the relationship among these events (Yang and Rudolf 2010).

## Models

### Simulations and nonmechanistic models

#### Overview

Simulations and nonmechanistic models are used primarily to generate testable predictions that address the third of our key questions: How will the phenological overlap of interacting species change (Fig. 1)? Parameter values can be adjusted to explore how various aspects of the phenology and behavior of species might influence interactions under different climate change scenarios. Models and simulations can also be extended to the community level (Nakazawa and Doi 2011); phenological shifts of varying magnitude and direction can be generated to investigate, for example, consequences for temporal overlap of interacting species (Memmott et al. 2007). Finally, theoretical models can provide insight into topics that are difficult to tackle empirically, such as how evolution may shape the phenological overlap of interacting species under future climate change (Gilman et al. 2012).

#### Examples

Recently, Fabina et al. (2010) used a theoretical approach to investigate how the phenological overlap among interacting species might change and what the consequences of two distinct phenological scenarios might be. The authors modeled interactions involving pollinators, herbivores, and a shared host plant to examine how the order of interactions affects the community. If climate change affects the phenology of the plant such that it is exposed to pollinators before herbivores, then the model predicts that the community will become destabilized, and the species can fail to coexist. If, on the other hand, changing climatic cues cause herbivores to be active before pollination occurs, then the predicted result is a higher density of plants and herbivores and a lower density of pollinators.

#### Merits

As is true for simulations and models in general, an advantage of this approach is its ability to explore the effects of variation in both biotic and abiotic parameters. Such flexibility in model parameterization can reveal unintuitive relationships and consequences that can generate novel hypotheses to be tested and explored empirically. Another advantage of a modeling approach is that multigenerational timescales can be easily investigated, which could be particularly valuable for species for which long-term data are unavailable or when studying evolution in real time is impractical.

#### Limitations

As with all models and simulations, attaining a realistic level of biological complexity can be challenging. Thus, the results, however general, may be difficult to apply to any particular system. Species-specific responses to climate change can be particularly difficult to model, as can indirect effects of altered phenological overlap of interacting species. In a similar vein, model results can depend strongly on the underlying assumptions, although this weakness can be alleviated by comparing results with and without assumptions of interest relaxed.

### Mechanistic models

#### Overview

Mechanistic models address the question: Why is the phenological overlap of interacting species changing (question 2, Fig. 1)? This approach is primarily concerned with identifying the mechanisms responsible for differential phenological shifts among interacting species. The relationship between phenological responses and climatic drivers is explored in mechanistic models, which can range from simple linear regression analysis to state-based models (e.g., Chuine 2000; Lambert et al. 2010). Unlike the models discussed previously, these are linked to the biological details of a specific system. Thus, detailed data on species' phenologies, as well as complementary data on the potential cues and triggers that dictate those phenologies, are required. The resulting models can then be used to quantitatively extrapolate into the future to ask how interactions are likely to respond to projected climate change (question 3, Fig. 1).

#### Examples

Many studies that have mechanistically modeled the phenologies of interacting species also employ an empirical approach (Table S1). Recently, Forrest and Thomson (2011) combined mechanistic models with an experimental manipulation. Reciprocal transplants of bees and wasps at two elevations provided data on emergence phenology. These were coupled with observational data on the flowering phenology of plants along an elevational gradient. Phenological and local temperature data were then used to parameterize models. Analysis revealed that although temperature affects the phenologies of both plants and pollinators, spring time warming is more likely to lead to earlier flowering than to earlier insect emergence. Thus, studies such as this address why the phenological overlap of interacting species is changing, and, by incorporating future climate scenarios, could address how the phenological overlap of interacting species will change.

#### Merits

Mechanistic models allow for more informed and more exact predictions of how the phenologies of individual species will respond to climate change and thus whether the phenological overlap of interacting species will shift. This mechanistic level of understanding is valuable, particularly if there is reason to believe that focal species are representative of their community. For example, white cabbage butterfly (*Pieris rapae*) phenology was used as a proxy for other insect pollinators of *Prunus* tree species based on evidence that they would respond similarly to changing climatic cues (Doi et al. 2008).

#### Limitations

It can be difficult to collect detailed phenological and high-resolution climatic data, especially for a large number of interacting species. Thus, mechanistic models trade-off precision for generality, as a model developed for one species in one location cannot be assumed appropriate for another. Furthermore, if the ultimate goal is to predict how interactions will change, mechanistic models may not be able to capture scenarios in which cues change beyond some unknown threshold or the correlations among cues are altered. For instance, a mechanistic understanding of a species' response to increased carbon dioxide levels may be limited to past and current levels unless an experiment is also conducted to determine the response under predicted, further elevated levels.

## Discussion

Our survey of the literature identified five approaches used in recent years to investigate the effects of climate change on the phenological overlap of interacting species and brought to light several key questions driving this work. We have pointed to unique advantages and disadvantages of each approach that dictate, in part, which key questions they can answer, individually or jointly. Disparities in the types of interactions receiving study became apparent, with most studies focusing on resource–consumer interactions, as did the underuse of certain approaches, such as short-term field observations.

Our analysis indicates that no single approach can answer all three of the key questions driving research on the phenological overlap of interacting species. Perhaps not surprisingly, 51% of the studies in our survey employed multiple methods, spanning an array of interaction types (Table S1). Many of these papers combined long-term data with experimental manipulations or simulations and nonmechanistic models (Table S1). With the use of several approaches in a single system, a more complete understanding of how phenological shifts will affect interactions can be achieved not only because multiple key questions can be answered (Fig. 1) but also because the limitations of one approach can be overcome by the strengths of another. For example, van Asch et al. (2007) used a three-pronged approach, combining long-term data, an experiment, and a simulation to study a plant–herbivore interaction. The authors used long-term data on dates of moth egg hatching and oak budburst, an experiment to determine moth fitness consequences of asynchronous hatching, and a simulated climate scenario to explore how egg hatching will respond to temperature under climate change. This study showed how the timing of this plant–herbivore interaction is changing and will change and also explored the consequences of change.

Although each combination of approaches has its own set of strengths and weaknesses, we specifically note here the value of combining long-term data with experimental manipulations. This combination has the ability to address all three key questions, in addition to addressing the consequences of phenological changes (Fig. 1). Moreover, a recent meta-analysis demonstrated the importance of long-term observational data to gauge whether experimental manipulations of phenology accurately reflect the effects of climate change (Wolkovich et al. 2012). Therefore, carefully designed experiments in combination with long-term data can provide a holistic picture of the effects of climate change on the phenological overlap of interacting species.

As the sole approach that can establish a baseline of interannual variation in the phenological overlap of interacting species, interpretation of long-term data is valuable for understanding both the ecological and evolutionary implications of phenological change. This baseline may be one of relative synchrony or asynchrony (Singer and Parmesan 2010), but without this knowledge, it is difficult to judge whether anthropogenic climate change causes temporal mismatches. Because some interactions are asynchronous by nature, finding evidence for contemporary asynchrony is insufficient cause for alarm. Unfortunately, long-term phenological data are relatively rare. Long time series on the phenologies of interacting species that coincide temporally and spatially are even rarer (Harrington et al. 1999). Although it is difficult to supplement historical, preclimate change data, existing data could be used more extensively and effectively (Primack and Miller-Rushing 2012). In addition, it is important to commence the collection of new data that can in the future provide insight into how interactions are changing along with the climate. A number of citizen science programs, such as Nature's Notebook, managed by the USA National Phenology Network (http://www.usanpn.org), have been initiated in recent years whereby the public can submit phenological observations, and many facilities, such as arboretums and botanical gardens, are collecting phenological data (Primack and Miller-Rushing 2009; Schwartz et al. 2012).

To better understand how ecological interactions are likely to be reshaped by phenological shifts, we need to expand our focus in several ways. Again, although long-term data can provide unique insight into how the phenological overlap of interacting species is changing, they must be combined with other approaches to project those changes into the future (Fig. 1). We advocate greater use of additional methods, such as experimental manipulations of phenology and mechanistic models that can improve our ability to anticipate future changes. Likewise, we advocate expanding our scope of inquiry to encompass a broader range of interactions. Data on more poorly studied systems should improve our ability to identify which kinds of interactions are most likely to be affected by phenological shifts, a critical conservation goal for the coming decades. An enlarged geographical range of research and documentation of phenological trends is also essential, especially for migratory species. Most work to date has been conducted in temperate settings (Table S1; Primack and Miller-Rushing 2011).

The consequences of phenological shifts can extend beyond interacting pairs of species to influence community- and ecosystem-level processes. Although ecological network theory and simulations can yield general predictions, we have very little empirical data about how phenological shifts will shape interspecific interactions at the community level (Diez et al. 2012; Encinas-Viso et al. 2012; but see Burkle et al. 2013), and less still about how altered interactions will scale-up to affect ecosystem processes and services (Hegland et al. 2009). Thus, studies that incorporate temporal reshuffling at these levels are likely to provide a wealth of new information.

Finally, phenological shifts are interacting with many other factors, including range shifts and landscape modification, to shape species interactions and community dynamics. There is concern that all these pressures together could cause a state shift on a global scale (Barnosky et al. 2012). Researchers have, however, made significant progress in a relatively short time in understanding the effects of climate change–induced shifts in phenology on individual species and their interactions. Going forward, a more balanced and deliberate use of the available approaches that reflects the importance of anticipatory knowledge will allow us to construct a more complete understanding of the ecological effects of climate change.

## Supporting Information

**Table S1**. Detailed results of a literature survey of studies that address the effects of climate change on the phenological overlap of interacting species. For the 82 studies we identified, we list the authors and year of publication, the title, the approach(es) used, the category of interaction (denoted −, − for competitive; +, − for antagonistic resource–consumer; and +,+ for mutualistic interactions), the specific type of interaction (e.g., plant–herbivore), the habitat (e.g., terrestrial), and the type of organism (e.g., vertebrate) studied.
